# Evaluation of Maternal Complications in Severe Preeclampsia in a University Hospital in Tirana

**DOI:** 10.3889/oamjms.2016.025

**Published:** 2016-02-24

**Authors:** Eriseida Ndoni, Redi Hoxhallari, Astrit Bimbashi

**Affiliations:** 1 *University Hospital of Obstetrics and Gynecology “Koço Gliozheni”, Tirana, Albania*; 2 *Italian Clinic “San Antonio”, Tirana, Albania*

**Keywords:** severe preeclampsia, eclampsia, HELLP syndrome, stroke, pulmonary edema, maternal outcome

## Abstract

**BACKGROUND:**

Preeclampsia is a hypertensive multisystem disorder of pregnancy that complicates up to 10% of pregnancies worldwide and is one of the leading causes of maternal and perinatal morbidity and mortality.

**AIM:**

To evaluate maternal complications associated with severe preeclampsia.

**METHODS:**

This is a retrospective cross-sectional study conducted in the UHOG “Koço Gliozheni”, in Tirana. Primary outcomes evaluated: maternal death, eclampsia, stroke, HELLP syndrome, and pulmonary edema. Secondary outcomes: renal failure, admission in ICU, caesarean section, placental abruption, and postpartum hemorrhage. Fisher’s exact test and Chi-squared test were used as statistical methods.

**RESULTS:**

In women with severe preeclampsia we found higher rates of complications comparing to the group with preeclampsia. Eclampsia (1.5% vs. 7.1%, P < 0.001), HELLP syndrome (2.4% vs. 11.0%; P < 0.001), stroke (0.5% vs 1.9%, P = 0.105) pulmonary edema (0.25% vs. 1.3%, P = 0.0035), renal failure (0.9% vs. 2.6%, P = 0.107), admission in ICU (19.5% vs. 71.4%, P = 0.007), caesarean section rates (55.5% vs. 77%, P = 0.508), placental abruption (4.3% vs. 7.8%, P = 0.103) and severe postpartum hemorrhage (3.2% vs. 3.9%, P = 0.628).

**CONCLUSION:**

Severe preeclampsia is associated with high rates of maternal severe morbidity and early diagnosis and timely intervention can prevent life treating complications.

## Introduction

Preeclampsia is a hypertensive multisystem disorder of pregnancy that complicates up to 10% of pregnancies worldwide and is one of the leading causes of maternal and perinatal morbidity and mortality [1]. Hypertension in pregnancy should be defined as a systolic blood pressure ≥ 140 mmHg and/or diastolic blood pressure ≥ 90 mmHg, based on the average of at least 2 measurements, taken at least 15 minutes apart, using the same arm [2]. The diagnostic criteria of preeclampsia used to be de novo hypertension after 20 weeks of pregnancy and proteinuria (exceeding 300 mg of total protein in a 24-hour urine collection) [3]. Recently, Royal College of Obstetricians and Gynecologists (RCOG) have proposed another mode of proteinuria assessment, using spot urine measuring the protein to creatinine ratio. According to the fact that preeclampsia is considered a multi-system disorder, the diagnostic criteria such as hypertension and proteinuria alone is not sufficient for clinical practice. Hence, this disease can be presented in several ways and it is necessary to asses all the symptoms and signs that are suggestive for the presence of the disease. The above mentioned facts has led to widening of the preeclampsia definition including: de novo hypertension after 20 weeks’ gestation and new onset of one of the following: a) proteinuria as defined above; b) renal insufficiency (creatinine > 0.09 mmol/L, or oliguria; c) liver disease (elevated transaminases and/or severe right upper quadrant or epigastric pain); d) neurological problems, convulsions (eclampsia), hyperreflexia with clonus, severe headaches, persistent visual disturbances; e) hematological disturbances: thrombocytopenia, DIC (Disseminated Intravascular Coagulation), hemolysis; or f) fetal growth restriction [4].

According to American College of Obstetrics and Gynecology (ACOG) diagnostic criteria, the diagnosis of severe preeclampsia includes severe hypertension (systolic blood pressure ≥ 160 mmHg or diastolic blood pressure ≥ 110 mmHg, or both), neurological disturbances (such as headache, visual disturbances, and exaggerated tendon reflexes), epigastric or right upper quadrant pain, oliguria (less than 500 mL in 24 hours), pulmonary edema, cyanosis, impaired liver function, thrombocytopenia or intrauterine growth restriction (IUGR) [5].

Preeclampsia and eclampsia account for 10–15% of maternal deaths worldwide [6]. A majority of deaths in developing countries result from eclampsia, meanwhile in developed countries result from the complications of preeclampsia (HELLP syndrome, eclampsia, DIC, renal failure, pulmonary edema) [7]. HELLP syndrome occurs in 0.1%-0.6% of all pregnancies and in 4%-12% of patients with preeclampsia. HELLP syndrome typically occurs between week 27 of gestation and delivery, or immediately postpartum in 15%-30% of cases [8, 9]. Eclampsia refers to the occurrence of new-onset, generalized, tonic-clonic seizures or coma in a woman with preeclampsia. Despite advances in detection and management, eclampsia remains a common cause of maternal morbidity and death.

Disseminated intravascular coagulation (DIC) was defined as platelet count < 100 000/mm^3^, plasma fibrinogen <3 g/L, and fibrin degradation products > 40 mg/dL [10]. The prevalence of DIC in pregnancy ranges from 0.03 to 0.35 percent in population-based studies [11].

Acute renal failure was diagnosed in the presence of oliguria or anuria in association with a creatinine clearance ≤ 20 mL/min or serum creatinine ≥ 2 mg/dL. Pulmonary edema is a rare and serious problem that complicates about 3 % of cases of severe preeclampsia [7].

The objective of this study is to evaluate major maternal complications associated with preeclampsia and severe preeclampsia.

## Material and Methods

This is a retrospective case control study conducted in the University Hospital of Obstetrics and Gynecology (UHOG) “Koço Gliozheni”, in Tirana. To collect the data for this study we used the medical records of deliveries from January 2009 until December 2013.

This study was approved by Institutional Review Board of UHOG “Koço Gliozheni”. Written informed consent was not obtained from pregnant women involved in this study, because it was a retrospective research and it was not possible to get the informed consent from every patient. The data collected for this study are anonymous.

The standard inclusion criteria in the study were pregnant women diagnosed with preeclampsia that had delivered in this hospital after 24 weeks gestation during the period mentioned above, despite the number of the babies, fetal presentation and mode of delivery.

The calculation of gestational age was made based on the first day of the last menstruation period (LMP – 13% of cases), on the early ultrasound examination (before 13 weeks gestation – 11% of cases) or based in the combination of both criteria (LMP and first ultrasound examination – 76% of cases).

The exclusion criteria in this study were: pregnancies with confirmed fetal lethal anomalies, pregnancies with missing data necessary for the study, pregnancies with inaccurate gestational age.

The variables collected from the medical records were: maternal age, parity, first day of the last menstruation (LPM), gestational age at the moment of severe preeclampsia diagnosis, gestational age at delivery, highest diastolic/systolic BP, medical treatment (antihypertensives, anticonvulsants, corticoids) as well as labor and delivery – spontaneous, induced or caesarean section (see Table 1).

**Table 1:** Maternal variables included in the study

Maternal variables
Maternal age
Parity
First day of the last menstruation (LPM)
Gestational age at the moment of severe preeclampsia diagnosis
Gestational age at delivery
Highest diastolic BP, Highest systolic BP
Highest proteinuria level
Medical treatment (antihypertensives, anticonvulsants, corticoids)
Labor and delivery – spontaneous, induced, caesarean section

The maternal outcomes measured were:

Primary outcomes that include: maternal mortality, eclampsia, stroke, HELLP syndrome (hemolysis, elevated liver enzymes and low platelets), and pulmonary edema. Pulmonary edema was assessed based on clinical findings and chest radiography.

Secondary outcomes that include: renal failure, admission in ICU (Intensive Care Unit), caesarean section, placental abruption, and postpartum hemorrhage. Renal failure was diagnosed when oliguria or anuria in association with an elevated serum creatinine level ≥ 2 mg/dL.

All the data were collected in excel format and were checked for their completeness and accuracy. The evaluated variables were compared between the preeclampsia group and the severe preeclampsia group.

The statistical analysis was made using SPSS program. Differences between groups for categorical variables were examined with Fisher’s exact test. In the situations with large numeric data of the variables we have used the “Chi-squared” test.

## Results

The total number of deliveries for this 5 years period (January 2009 until December 2013) was 21,795. After a careful investigation of medical records, were identified 1274 cases hospitalized with hypertensive disorders, of which 897 were diagnosed with preeclampsia.

Based on the exclusion criteria we found 27 pregnancies with confirmed fetal lethal anomalies, 99 pregnancies with missing data necessary for the study (in the medical records there were not all the available data for the variables included in the study), 28 pregnancies with inaccurate determination of gestational age (the patient doesn’t know the LMP and/or hasn’t done an ultrasound examination in the first trimester).

After the application of the exclusion criteria, a total of 743 cases with preeclampsia were included in this study. Based on ACOG classification criteria (2013) for the severity of the diseases we found 154 cases with severe preeclampsia (0.7% of the total births of the study period and 20.7% of all cases with preeclampsia).

Taking into consideration the fact that our study extends in a 5-year period, the population available to this study do not allow for analysis of maternal mortality. For this reason, regardless of maternal mortality is considered as the most important parameter in the evaluation of maternal outcome, referring to the small sample of this study, it was impossible the statistical processing of the results on this variable.

Eclampsia and HELLP syndrome are considered almost exclusively as unique complications of preeclampsia. For this reason the number of cases with eclampsia and HELLP syndrome is equal in both preeclampsia and severe preeclampsia groups.

### Primary maternal outcomes

In Table 2 are presented the major maternal complications in cases with preeclampsia and severe preeclampsia (primary outcomes) and comparison was done between the two the groups for all the outcomes. In this comparison we noted a statistically significant difference for the presence of eclampsia (1.5% vs. 7.1%, for total preeclampsia group vs. severe preeclampsia group respectively; *P* < 0.001). The same significance was observed even for HELLP syndrome (2.4% vs. 11.0%; *P* < 0.001). Stroke and pulmonary edema were more frequent in the severe preeclampsia group but this difference was not statistically significant (*P* = 0.105 and *P* = 0.0035 respectively).

**Table 2:** Primary maternal outcomes in severe preeclampsia and in total cases with preeclampsia

Primary maternal outcomes	Total preeclampsia n (%)	Severe preeclampsia n (%)	*P* value
Eclampsia	11/743 (1.5%)	11/154 (7.1%)	*P* < 0.001
HELLP syndrome	18/743 (2.4%)	18/154 (11.0%)	*P* < 0.001
Stroke	4/743 (0.5%)	3/154 (1.9%)	*P* = 0.105
Pulmonary edema	2/743 (0.25%)	2/154 (1.3%)	*P* = 0.0035

### Secondary maternal outcomes

In Table 3 are presented the rates of secondary outcomes for both groups. In the comparison between the two groups we noted a statistically significant difference for the admission in ICU (19.5% vs. 71.4%, for total preeclampsia group vs. severe preeclampsia group respectively; *P* = 0.007).

**Table 3:** Secondary maternal outcomes in severe preeclampsia and in total cases with preeclampsia

Secondary maternal outcomes	Total preeclampsia n (%)	Severe preeclampsia n (%)	*P* value
Renal failure	7/743 (0.9%)	4/154 (2.6%)	*P* = 0.107
Admission in ICU	145/743 (19.5%)	110/154 (71.4%)	*P* = 0.007
Caesarean section	413/743 (55.5%)	118/154 (77%)	*P* = 0.508
Placental abruption	32/743 (4.3%)	12/154 (7.8%)	*P* = 0.103
Severe PPH*	24/743 (3.2%)	6/154 (3.9%)	*P* = 0.628

* Severe Postpartum hemorrhage (> 1000 mL).

The rate of renal failure resulted higher in women with severe preeclampsia than in women with preeclampsia, but the difference was not significant (0.9% vs. 2.6%, *P* = 0.107). The same results we had even for caesarean section rates (55.5% vs. 77%, *P* = 0.508), placental abruption (4.3% vs. 7.8%, *P* = 0.103) and severe postpartum hemorrhage (3.2% vs. 3.9%, *P* = 0.628).

## Discussion

Pre-eclampsia constitutes a major source of morbidity and mortality worldwide. Overall, 10%–15% of maternal deaths are directly associated with severe preeclampsia and eclampsia [6]. Although maternal mortality is a very important parameter in the evaluation of maternal outcome, referring to the small sample study, we considered impossible the statistical processing of the results on this variable.

Maternal complications generally correlated with the severity of preeclampsia. In our study we found that 1.5% of all preeclamptic women and 7.1% of all severe preeclamptic women were complicated by eclamptic seizures. In a previous study, Liu et al. demonstrated that 9.34% of cases with severe preeclampsia had eclampsia [12], which is slightly higher than our results.

Our study showed that, 2.4% of cases with preeclampsia and 11% of cases with severe preeclampsia had HELLP syndrome. Also Murphy and Stirrat found that 21% of cases with severe preeclampsia had HELLP syndrome which is almost the double of our result (21% vs. 11%) [13].

In our study we found stroke in 0.5% of cases with preeclampsia and 1.9% of cases with severe preeclampsia. Comparing to the literature in the PIERS study CNS complications were found 7 from 2020 cases with preeclampsia (0.35%), similar to our result for the preeclampsia group [14].

As well, our study we found pulmonary edema in 1.3% of cases with severe preeclampsia. This result is higher than the results found by Yildirim et al. (5/903 cases, 0.6%) [15], and lower than the results found in Tuffnell et al. (25/1087 cases, 2.3%) [16]

In our study cesarean delivery rate in severe preeclampsia group was 77 %. Similar to our study we found increased rates of caesarean section in even in the studies of Liu et al. [12 Error! Reference source not found.] and Murphy and Stirrat [13] (87.3 and 80 % respectively). In our study 7.8 % of cases with severe preeclampsia presented placental abruption which is less than the rate of 15% reported by Murphy and Stirrat [13].

Yildirim et al. in their study found renal failure in 14 of 903 cases with severe preeclampsia (1.6%) while in our study we found renal failure in 4 of 154 cases (2.6%) [15].

We concluded that severe preeclampsia is associated with high rates of maternal severe morbidity. Early diagnosis and timely intervention can prevent life threating complications from this disorder and can improve maternal outcomes.
